# “Roberto Rodríguez” General Teaching Hospital of Moron, Ciego De Avila, Cuba, Neurosurgery and Pediatric Intensive Care Services Pediatric Neuromonitoring in Severe Head Trauma

**DOI:** 10.1089/neur.2024.0031

**Published:** 2024-05-29

**Authors:** Daysi Abreu Pérez, Angel J. Lacerda Gallardo, Jose Antonio Gálvez

**Affiliations:** ^1^ Department of Pediatric Intensive Care Unit, “Roberto Rodríguez Fernández” General Teaching Hospital of Morón, Ciego de Ávila, Cuba.; ^2^ Department of Neurosurgery, “Roberto Rodríguez Fernández” General Teaching Hospital of Morón, Ciego de Ávila, Cuba.; ^3^ Department of Neurosurgery, General Teaching Hospital of Morón, Ciego de Ávila, Cuba.

**Keywords:** neuromonitoring, severe head trauma, treatment of severe head trauma

## Abstract

Among all types of trauma in children, traumatic brain injury has the greatest potential for the development of devastating consequences, with nearly three million affected each year in the world. A controlled, nonrandomized experimental study was carried out in pediatric patients with severe traumatic brain injury, whose objective was to evaluate the use of continuous multimodal neuromonitoring (MMN) of intracranial parameters as a guide in the treatment of children of different age-groups. The patients were divided into two groups according to the treatment received; clinical and imaging monitoring was performed in both. Group I included those whose treatment was guided by MMN of intracranial parameters such as intracranial pressure, cerebral perfusion pressure, and intracranial compliance, and group II included those who had only clinical and imaging monitoring. Eighty patients were studied, 41 in group I and 39 in group II. There were no significant differences between the groups with respect to the sociodemographic variables and the results; as a consequence, both forms of treatment were outlined, for patients with MMN and for those who only have clinical and imaging monitoring. It is concluded that both treatment schemes can be used depending on technological availability, although the scheme with MMN is optimal.

## Introduction

The greatest challenge for medical personnel caring for pediatric patients affected by severe traumatic brain injury (sTBI) is to avoid or minimize secondary brain damage.^1,2^ These are dynamic and complex changes that occur in brain physiology and biochemistry, produced by hypoxia and hypotension, which trigger the development of edema, as the beginning of a cascade that generates intracranial hypertension (ICHT), reduction in cerebral blood flow, limited oxygenation, and, as a consequence, poor results.^1,2^

Among all types of trauma in children, traumatic brain injury (TBI) has the greatest potential for the development of devastating consequences, with nearly three million affected each year in the world.^3^ It is also the main cause of disability and death worldwide at these ages, regardless of the cause. Traffic accidents are the most common cause of TBI in all ages and are estimated to be the seventh cause of death by 2030.^3^

The frequency of sTBI in pediatrics is highly variable, the numbers are usually lower in developed countries compared with underdeveloped or developing countries, with a global annual incidence of emergency department visits of 691/100,000, hospitalizations amounting to 74/100,000, and deaths at 9/100,000.^4,5^

In Cuba, sTBIs are closely related to accidents and constitute the fifth cause of death in adults and the first in those under 45 years of age,^6^ but there are no national rates on the incidence and prevalence in the pediatric population. In previous experiences published by our group, it was found that only 2.39% of all head injuries in children were serious, with a mortality for this subgroup of 31.25%.^7^

ICHT is a complication frequently associated with these cases, and the time of exposure of brain tissue to high levels of intracranial pressure (ICP) is directly related to unfavorable results,^8^ so its monitoring and control are essential. Advances in multimodal neuromonitoring (MMN) of intracranial physiology and, correspondingly, neurointensive treatment have facilitated the understanding and identification of optimal therapeutic targets after the traumatic event has occurred in the adult patient; however, these strategies should not be applied to the pediatric population, and consequently, its benefits are still debated in the world scientific community.^9,10^

For some decades now, there has been sufficient evidence that the ICP doesn’t reflect the entire clinical picture, especially if the patient is in a state of sedation or neuromuscular blockade and artificial mechanical ventilation (AMV),^11,12^ so the MMN is presented as an essential source of information for the neurointensivist, such as cardiovascular or respiratory monitoring for other subspecializations within intensive care; however, this is an aspect that is currently controversial despite the fact that it is known that ICHT events without clinical translation represent a serious risk factor for mortality.

The lack of consensus in the international scientific community on the benefit of MMN as a guide for the treatment of pediatric patients suffering from sTBI and the lack of knowledge of the optimal thresholds of ICP and cerebral perfusion pressure (CPP) in children of different ages demands the design and execution of clinical studies that allow obtaining the necessary evidence on the subject and facilitate the use of appropriate therapeutic measures to guarantee the necessary balance between the structures contained in the intracranial compartment.

The Latin American consensus meeting, held in Buenos Aires in 2015 and the proposal of the CREVICE, has represented an option for those patients with sTBI who, for different reasons, do not receive MMN during their treatment.^13^

The child is not a small adult; there are anatomical and physiological differences between pediatric age-groups and between children and adults, which must be considered to guarantee optimal treatment adjusted to each age-group.^14^ The guidelines show that there isn’t enough evidence^12,15,16^ on the optimal ICP and CPP thresholds to initiate the treatment of ICHT in childhood, which is extremely controversial.^12,15,16^

In Cuba, the information available on the subject is scarce, and the experience of the working group of the “Roberto Rodríguez Fernández” General Teaching Hospital of Morón, Ciego de Ávila, stands out.^17–19^

Based on this, the following research problem arises: Will the contribution of MMN be superior to clinical and imaging monitoring as a guide for the optimal treatment of pediatric patients with sTBI?

With the hypothesis that continuous neuromonitoring of ICP, CPP, and intracranial compliance will contribute to obtaining better results at discharge in pediatric patients with sTBI, we proposed the following objective: to evaluate the use of continuous MMN of intracranial parameters as a guide in the treatment of children of different age-groups who suffer from a TBI.

## Method

A controlled, nonrandomized experimental study was carried out with all patients admitted to the pediatric intensive care unit (PICU) with a diagnosis of sTBI, Glasgow Coma Scale (GCS) of 8 points or less, in the period between 2000 and 2020.

There were two groups. In group I, 41 patients were included, to whom a diagnosis and treatment algorithm based on clinical and imaging neuromonitoring was applied, to which MMN was added. In group II with 39 cases, a diagnosis and treatment algorithm guided only by clinical and imaging monitoring was applied. For this process, the medical records of the patients were reviewed and the information was extracted, according to the algorithm proposal arising from the “Buenos Aires Consensus” on the treatment of sTBI in patients without invasive neuromonitoring, held in 2015, which was attended by one of the authors of this study (A.J.L.G.) and which was published in 2020.^13^ The data of the patients included in this group and who were treated before 2015 were adjusted to the requirements of CREVICE modified by the authors. The constitution of the groups was not randomized.

All cases were evaluated and studied in a medical emergency and were then admitted to the pediatric intensive care service.

### Evaluation of the algorithms before the quasi-experiment

In the first phase of the study, the work algorithms were designed or modified for both groups of patients; those of group II were adjusted to the characteristics of the pediatric patients, taking as references to those proposed by CREVICE,^13^ which were presented to the neurosurgery and pediatric intensive care groups, to submit them to expert judgment, according to the qualitative technique of the nominal group.^20^

### Nominal group structure

The nominal group was made up of 12 specialists with sufficient experience in the treatment of pediatric sTBI who work in the neurosurgery and pediatric intensive care services.

For characterization, they underwent a self-assessment of their expertise in the diagnosis and treatment of sTBI in pediatrics. The information was obtained through a form to identify the following coefficients in each of the members: Knowledge coefficient (Kc): calculated by multiplying the selected number on a scale of 1 to 10 by 0.1.

Argumentation coefficient (Ka): sum of the values obtained in the standard table. Competition coefficient (Kcp): It was calculated by the formula: ½ (Kc + Ka).

- If 0.8 < Kcp < 1.0: the coefficient is high.
- If 0.5 < Kcp < 0.8: the coefficient is medium.
- If Kcp < 0.5: the coefficient is low.

At the end of the debate, 100% of the members of the nominal group considered that the algorithms were adequate.

### Inclusion criteria:

- Age from 1 month of birth to 17 years 11 months and 29 days.
- TBI with GCS of 3–8 points.
- Patients whose parents or guardians offered their consent to participate in the research and signed the document, after having received information about the characteristics of the study.

### Exclusion criteria:

- Transfer of the pediatric patient to another hospital owing to associated injuries.
- Nonacceptance of parents or guardians of the patient to participate in the study.

### Criteria for MMN:

- Abnormal computarized tomography (CT) on admission.
- Patient with normal CT and presence of unilateral or bilateral motor postural alterations, and
- Systolic blood pressure (SBP) below the 5th percentile for each age-group.

### Clinical monitoring

Continuous clinical monitoring was carried out in both groups by the group of pediatric intensivists and neurosurgeons, 24 h a day, which was characterized by cardiorespiratory, hemodynamic, hydromineral and acid–base balance, and hemogasometric monitoring. The clinical examination included the evolutionary neurological profile of the patients at least every 4 h, with which the level of consciousness was observed in cases that allowed it, the pupillometry and pupillary reactivity, the respiratory and hemodynamic pattern, the motor pattern, and the level of cephalocaudal degradation of the patient as an integrator of the neurological state.

### Imaging monitoring

This was carried out in both groups. CT studies were performed on admission and sequentially after 12 h of evolution if the first CT was obtained in the first 4 h of evolution of the trauma. New studies were indicated after 24 h and 48 h of evolution. They were also urgently indicated in the event of any neurological deterioration or sustained elevations in ICP above the values considered for each age-group for 15 min despite the use of measures to control ICP.

Evaluation of CT images on admission was performed according to the classification of Marshall et al.^21,22^ and was individualized by the neurosurgeon on duty. The evolutionary CT scans were discussed by a group of specialists made up of neurosurgeons, neuroimaging specialists, and pediatric intensivists, with the aim of obtaining a collegiate diagnosis and consequently adopting the treatment.

The sequenced CT images were classified as “improved” (total return or more than 50% of the initial measurement of the dislocation of the midline structures, reduction of cerebral edema with reopening of the diameters of the ventricular system, reopening of the cisterns, and reduction of more than 50% of the thickness and total volume of lesions occupying intracranial space), “unchanged” (when there were no significant changes with respect to the images acquired upon admission), and “worse” (when the images acquired on admission worsened or new lesions appeared).

The volume of the focal lesions was calculated according to the ellipsoid law (A × B × C/2 where A = greatest length, B = greatest width, and C = height).^23^ The diameter of the optic nerve sheath (ONSD) was also measured in the initial and subsequent CT scans. This measurement was also performed with orbital ultrasound in the PICU, at the patient’s bedside. The measurement was made 3 mm behind the eyeball transverse to the optic nerve.

Simple chest X-rays of long bones, rib cage, and bony pelvis were indicated for patients with injuries at these levels. Abdominal ultrasounds and transcraniectomy were indicated at medical discretion in selected patients.

### Description of the algorithms

These were designed based on the algorithms used by the pediatric intensive care and neurosurgery services since 1996, which were based on the guidelines for the acute medical management of sTBI in infant, children, and adolescents and its update.^12,15,16^ These basic algorithms were modified based on a review of the state of the art on the subject and studies carried out by the authors to adjust them to the physiological characteristics of children at different moments in their evolutionary development.^7,12,13,15–19,24^

**FIG. 1.**
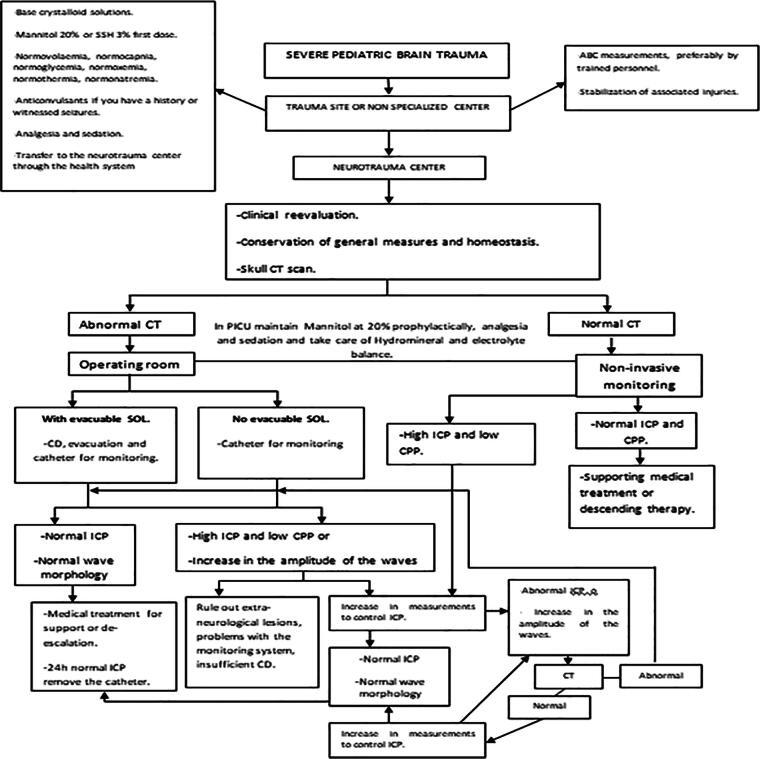
Work algorithm for pediatric patients with MMN. MMN, multimodal neuromonitoring.

The diagnosis and treatment algorithm for patients with MMN begins from the trauma site or previous phase to the neurotrauma center (Fig. 1). The measures to control ICP were organized into three levels (Table 1) so that they were introduced in an additive and staggered manner, although, depending on the therapeutic needs and technological availability, they may not follow the order in which they are described within the same level and even between different levels. The downscaling or withdrawal of the measures was carried out in the same order in which they were introduced, depending on the clinical symptoms, the neuroimaging, and the information on the different MMN variables.

**Table 1.** Treatment Levels for ICHT

Level 1	CSF drainage. Five milliliters up to four times in 1 h. Twenty percent Mannitol regimen in boluses at 0.25–0.50 g/kg/4 h. 3% Hypertonic Saline Solution Scheme in boluses at 1–2 mL/Kg/4 h. Maintain normothermia with a threshold of 37.5°C. Primary decompressive craniectomy.
Level 2	Deepen sedation. Moderate hyperventilation PaCO_2_ ≥ 30–35 mmHg. 3% Hypertonic Saline Solution in continuous infusion. Neuromuscular blockers.
Level 3	Delayed or secondary decompressive craniectomy. Barbiturate coma. Lumbar CSF drainage. Surface hypothermia with threshold < 37°C.

ICHT, intracranial hypertension.

**FIG. 2.**
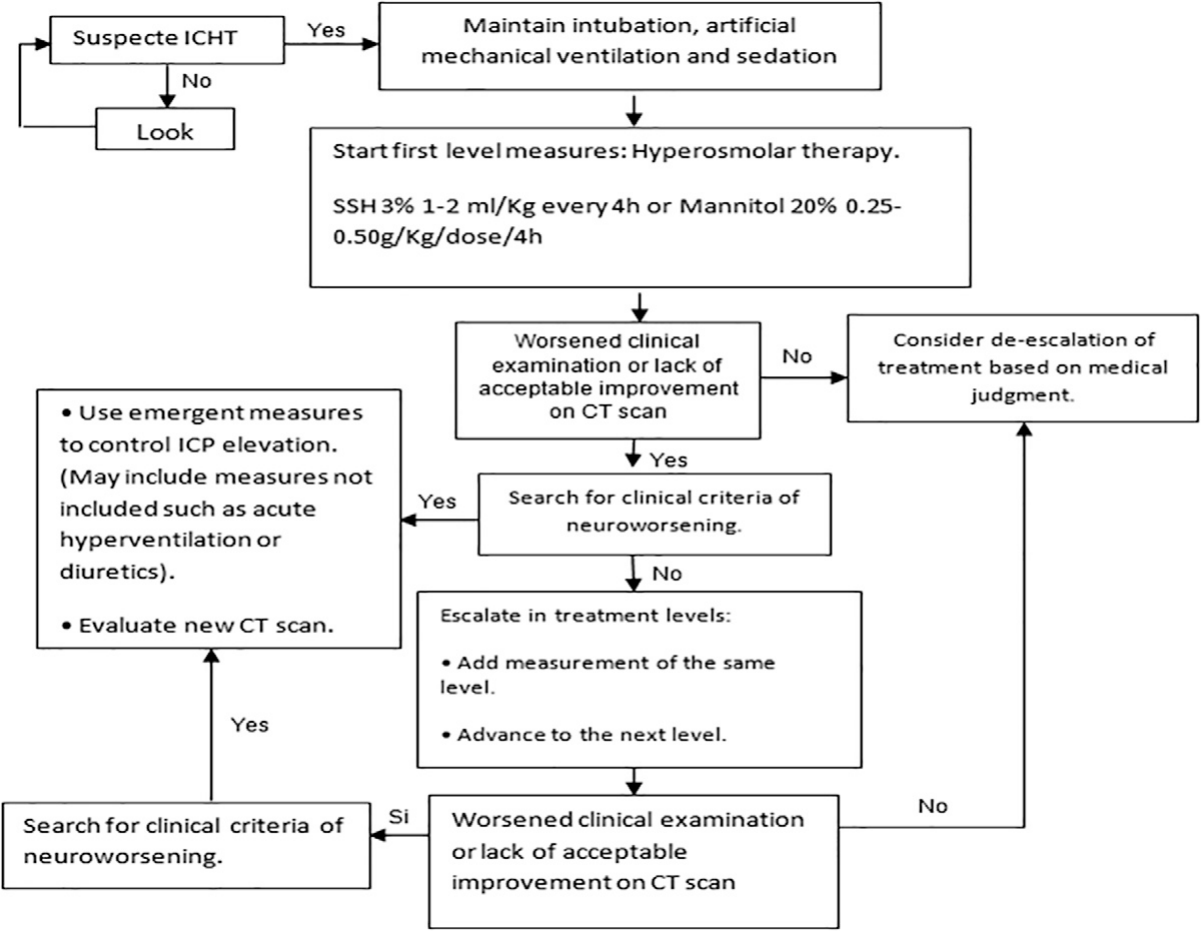
Work algorithm for pediatric patients with clinical and imagenological monitoring.

The diagnosis and treatment algorithm for patients without MMN was based on the CREVICE clinical and imaging monitoring protocol, which has its origins in the Buenos Aires consensus, carried out in 2015 and published in 2020^13^ (Fig. 2), to which modifications were made to adjust it to the objectives of the present study with pediatric patients.

ICHT was clinically suspected, and treatment was initiated in the presence of one major criterion or two minor criteria.^13^

Major criteria: 1—compressed cisterns (Marshall Classification III) on CT; 2—midline dislocation ≥ 5 mm (Marshall Classification IV) on CT; 3—lesion with mass effect not evacuated on CT.

Minor criteria: 1—Glasgow Motor Scale ≤ 4; 2—pupillary asymmetry; 3—abnormal pupillary reactivity; 4—Marshall Grade II diffuse axonal injury (cisterns present with 0–5 mm dislocation or focal lesions of high density or mixed less than or equal to 25 cm^3^).

If there is suspicion, the patient is kept intubated, with AMV, baseline sedation, and first-level treatment measures are initiated (Table 1). An essential objective was to achieve and maintain a mean arterial pressure (MAP) ≥ 5th percentile for each age-group.

If the clinical examination or CT scan is stable or shows signs of acceptable improvement, therapeutic de-escalation is considered according to the specialist’s criteria. In this scheme, frequent neurological examinations by the medical team are required.

Neuroworsening was considered when any of the following findings appeared during the physical examination: 1—decrease of more than one point on the Glasgow Motor Scale; 2—new loss of pupillary reactivity; 3—appearance at intervals of pupillary asymmetries of more than 2 mm of difference or bilateral mydriasis; 4—appearance of a new motor focus; and 5—presence of a brain herniation syndrome (e.g., Cushing’s triad).^13^

Variables such as age, sex, origin, transportation used for transfer, time between the trauma and arrival at the neurotrauma center, treatment in the phase prior to the neurotrauma center, clinical and imaging diagnoses upon admission were studied, the presence of neuroworsening, the characteristics of the sequenced CT scans, the GCS at admission and its relationship with the GOS, and the quantitative values of the ICP and its relationship with the qualitative characteristics of the pulse waves were evaluated. We also evaluated CPP in group I and intracranial compliance as well as the treatments used in the PICU, complications, and results.

To personalize the treatment according to the different age-groups, the anatomical and physiological characteristics that distinguish children in different age-groups and adults were considered.

### Systemic blood pressure

Those who had figures lower than the fifth percentile for all ages were considered hypotensive (SBP: <65 mmHg for infants, <78 mmHg for 1-year-old children, <84 mmHg at 5 years, <88 mmHg at 10 years, and <98 mmHg over 14 years of age); (diastolic blood pressure [DBP]: <40 mmHg in infants and children aged 1 year, <44 mmHg at 5 years, <52 mmHg at 10 years, and <60 mmHg over 14 years of age).

The normotensives were those who showed figures between the 25th and the 75th percentile (SBP: 84–100 mmHg in infants and at 1 year, between 92 and 104 mmHg at 5 years, between 100 and 116 mmHg at 10 years, and 110–124 mmHg in those over 14 years of age); (DBP: between 50 and 62 mmHg in the infant and at 1 year, between 55 and 62 mmHg at 5 years, between 66 and 74 mmHg at 10 years, and between 70 and 80 mmHg in 14 years of age or older).

Hypertensive patients presented values higher than the 90th percentile (SBP: 100 mmHg in infants and at 1 year, >108 mmHg at 5 years, >120 mmHg at 10 years, and >132 mmHg in children over 14 years of age); (DBP: >67 mmHg in infants and 1-year-old children, >68 mmHg at 5 years, >80 mmHg at 10 years, and >70 mmHg in those over 14 years of age). From these values, the MAP is calculated through the formula two times the diastolic pressure plus the systolic pressure over three.

### Particularization of the ICP

Patients were divided according to normal ICP values in each age-group, taking the maximum value for evaluation. Infants: normal values between 3 and 6 mmHg, divided into (a) less than 6 mm of mercury and (b) ≥6 mm of mercury.^25^

Children over 1 year of age: normal values 7–15 mmHg, as the pressure range is so wide, as well as the age range, we divided this group into two subgroups (the existence of buffer mechanisms was considered up to approximately 4 years of age). In children 1–4 years old, the normal value is 10 mmHg; it is divided into (a) less than 10 mmHg and (b) 10 or more mmHg. For children between 5 and 17 years 11 months and 29 days, the normal value is 15 mm of mercury, and they were divided into (a) less than 15 mmHg and (b) ≥15 mm of mercury.^25^

ICHT was treated when the values were higher than the upper normal value for each age-group, with the exception of those who presented lesions occupying intracerebral space, located in the uncus of the temporal lobe or in the basal frontal region, in which the therapeutic behaviors were evaluated individually.

Specification of the CPP:

CPP was calculated according to the conventional formula CPP = MAP − ICP, where MAP is the mean arterial pressure obtained in turn by the formula to perform the calculation.

As MAP values vary according to age-groups and its normal range fluctuates between the 5th and the 90th percentile, and, furthermore, ICP values also vary according to age; to calculate this variable, the following values were used: minimum MAP and maximum ICP values for each age-group. Optimal values were considered.^25^

- For infants and children between 1 and 4 years old, 47 mmHg, so the results were divided into (a) less than 47 mmHg and (b) 47 or more mmHg.
- For children between 5 and 17 years 11 months and 29 days, the normal figure is 50 mmHg, so they were also divided into (a) less than 50 mmHg and (b) 50 or more mmHg.

For patients with normal first CT, noninvasive monitoring with carotid Doppler, transcranial Doppler, head CT, transcraniectomy ultrasound, and ONSD by transorbital ultrasound or CT are indicated. To estimate ICP and CPP, data obtained from transcranial Doppler are used with the application of the formula: ICP = MAP − CPPe, where CPPe = MAP × Vd/Vm^−1^ + 14. The flowchart is inserted in the variants described previously according to the quantitative values and qualitative characteristics of the ICP and CPP waves.

### Particularization of intracranial compliance

This variable was determined in the group with invasive ICP monitoring, using the pressure volume index (PVI), which was calculated by adding CSF volume or subtracting it. The applied technique was selected depending on the ICP at the time of the calculation; if the ICP was normal or below the normal value, it was performed by addition, and if the ICP was elevated, it was performed by subtraction to avoid unwanted elevations and the generation of possible pressure cones.

Calculation by addition:

$$\text{IPV}=\frac{\text{Added volume}}{\text{Log10}\frac{\text{PF}}{\text{PI}}}$$

Where: PI is the ICP recorded by the monitor before the injection of the solution and PF is the ICP recorded by the monitor after injection of the solution. When the calculation was carried out by subtraction, the values of the initial and final pressures were inverted, so the formula was as follows:

$$\text{IPV}=\frac{\text{Volume stolen}}{\text{Log10}\frac{\text{PI}}{\text{PF}}}$$

Log10=1

To calculate the normal ranges of PVI by adding 1 mL of solution, it is necessary to know the normal permissive upper ICP value by age-group, and to this value, add and subtract 4, which is the normal permissive value of elevated ICP in mmHg before the injection of 1 mL of solution into the intracranial cavity.

The distribution of the normal ranges of PVI by age would be as follows: For those under 1 year of age with permissive upper normal ICP at 6 mm of mercury (6 ± 4 mmHg), the normal IPV would be in the range between 0.33 and 0.60 mL. For children older than 1 year in which the normal ICP is between 7 and 15 mmHg, it would be 7 ± 4 and 15 ± 4 mmHg, the normal IPV is between 0.63 and 0.79 ml. For the subgroup of children between 1 and 4 years of age in whom the normal permissive upper ICP value is 10 mmHg (10 ± 4 mmHg), the normal IPV would be in the range between 0.60 and 0.71 mL.

For the age subgroup between 5 and 17 years in which the upper normal ICP value is 15 mmHg, then it would be 15 ± 4 mmHg, and the normal PVI is between 0.74 and 0.79 mL.

The data were obtained from the medical records and were entered into a database built with the SPSS version 21.0 system for Windows. The no parametric Mann–Whitney *U* tests were applied to compare two sample means and the Kruskal–Wallis to compare medians. We also used Fisher’s exact test to relate two quantitative variables.

## Results

Table 2 shows the general characteristics of both groups, which shows that there was a predominance in both cases between 5 and 17 years of age, the transportation used mostly was health care, and the time period between the moment of the accident and arrival at the neurotrauma center was predominantly between 0 and 3 h.

**Table 2.** General Characteristics

*General variables*	*Category*	*Group I*			*Group II*		
		*No.*	*%*	^a^P	*No.*	*%*	^a^P
Age	Less than 1 year	2	4.9	0.000			0.000
	1–4 years	4	9.8		3	7.7	
	5–17 years	35	85.3		36	92.3	
Sex	M/F	28/13	65.1/30.2	0.019	28/11	71.8/28.2	0.021
Area of origin	South	23	56.1	0.435	27	69.2	0.398
	North	18	43.9		12	30.8	
Transport used	Sanitary	37	90.2	0.000	36	92.3	0.000
	No Sanitary	4	9.76		3	7.7	
Time to arrival at the neurotrauma center	0–3 h	29	70.7	0.000	31	79.5	0.000
	4–6 h	9	22		8	20.5	
	7–12 h	3	7.3				

^a^ Pearson chi-square.

In the phase prior to the neurotrauma center, most of the children had received a dose of brain dehydrators and had been resuscitated with crystalloid-type fluids (0.9% physiological saline solution).

In the clinical diagnoses upon admission, there was a predominance of moderate diffuse axonal damage, 20 patients (48.8%) in group I and 13 cases (33.3%) in group II.

The GCS upon admission and its relationship with the GOS showed that in both groups, patients with GCS upon admission predominated in eight points, 21 cases (51.2%) in the group I, of which 18 (85.7%) were left with mild or no sequelae and 22 (56.4%) in group II, of which 17 (77.3%) were left with light or no sequelae. In both groups, all the cases admitted with GCS at three points died.

In group I, 9 patients (22%) presented neuroworsening criteria, whereas in group II, there were 18 cases (46.1%), with a difference of 24.1% in favor of group II. The use of CT upon admission was a decisive element in the application of the algorithms. In group I, grade IV diffuse injury (shift) predominated, with 18 cases (43.9%) and grade III (swelling) with 12 patients (29.3%). In group II, 17 cases (43.6%) showed diffuse injury type IV and 14 cases (35.9%) showed grade III.

When comparing the status of the structures in the midline in the CT at admission between the groups, it is evident that in both there was a relationship between dislocations of 0–10 mm with those discharged alive, whereas patients with dislocations greater than 10 mm were related to the deceased. When applying the Mann–Whitney *U* test, a correlation was found between these two variables with a significance level of *p* ≤ 0.03 in group I and *p* ≤ 0.04 for group II. Furthermore, in group I, compressed cisterns were more frequent with 20 cases (48.8%), of which 18 (90%) were discharged alive and absent cisterns in 18 patients (43.9%), of which 10 (55.6%) died. In group II, 25 cases (64.1%) had absent cisterns, of which 14 (56%) died and 14 patients showed compressed cisterns (35.9%), all of whom survived.

ONSD measured on CT at admission were homogeneous in both groups, with a greater frequency of those with 3.5 mm or less, 21 (51.2%) in group I and 18 (46.2%) in group II. When relating the ONSD with the average ICP values in group I, it was striking how 24 (85.7%) of 28 cases in the range between less than 3.5 mm and 5.5 mm had normal ICP values and 12 (92.3%) of 13 children with diameters greater than 5.5 mm had high ICP values for their age. When applying the Mann–Whitney *U* test, we found a correlation between ONSD and ICP for a significance level of *p* ≤ 0.000.

Continuous monitoring of ICP in group I showed that in the first 24 h of starting treatment, 25 patients (61%) had normal values for their age, whereas the number of patients with normal ICP increased between the second and on the fifth day of evolution to 33 cases (80.5%). In group II, as there was no MMN, the clinical and imaging estimation of the existence of ICHT was used, which is less reliable, so all patients in group II had suspicion of ICHT in the first 24 h, whereas in the following 25–120 h of evolution, only 16 patients (41%) showed normal ICP criteria, with a difference between the groups of 39.5%. CPP was the second most important variable considered in group I. In the study, a predominance of normal CPP values was observed in 27 cases (65.9%), which correspond to the predominance of normal CPP values and ICP from the first 24 h after starting treatment. When relating CPP to the status at discharge, we found that 11 (91.7%) of the 12 deceased patients had decreased CPP and 26 (89.7%) of the living discharged patients had normal CPP. By applying Fisher’s exact statistic, a significant result is obtained (*p* ≤ 0.000), which indicates that exposure to a decreased CPP is associated with deceased discharge and that decreased CPP behaves as a risk factor that increases 21.2 times the risk of dying in those exposed to this characteristic compared with patients with normal CPP.

Regarding intracranial compliance, a discrete predominance of patients with “loose” compliance and its significant correlation with normal ICP was observed. “Compromised” compliances were second in frequency and were related to increased ICP. Normal compliances were the least frequent, in which there was 50% shared representation between elevated and normal ICPs. If we combine the “normal” ones with the “loose” ones, we would reach 24 (58.5%), a little more than half of the patients. By correlating intracranial compliance with the characteristics of the ICP pulse waves, we observed that 76% of the patients with normal ICP had normal pulse wave morphologies, whereas 62.5% of the cases with elevated ICP had altered pulse wave morphologies. No complications occurred during the procedures to calculate PVI.

In the clinical evolution in the intensive care unit, we observed that in group I nine patients (22%) presented criteria for neuroworsening: three of them (33.33%) had a decrease in the Glasgow Motor Scale, three (33.33%) showed a new loss of pupillary reactivity, two (22.23%) presented pupillary asymmetries at intervals, and one (11.11%) had a new motor focus. In group II, 18 cases (46.1%) showed neuroworsening, with a difference of 24.1% in favor of group II. Six (33.33%) had a decrease in the Glasgow Motor Scale, three (16.67%) had a new loss of pupillary reactivity, four (22.22%) had pupillary asymmetries at intervals, and two (11.11%) and three (16.67%) showed brain herniation.

**FIG. 3.**
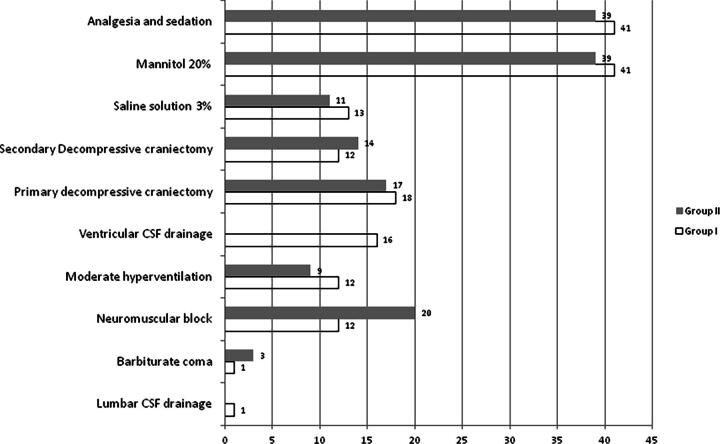
Treatment used.

Figure 3 shows the treatment used in the neurotrauma center. It is striking how analgesia and sedation and 20% mannitol were used in 100% of the cases in both groups. Primary decompressive craniectomy (DC) was used in 18 patients (43.9%) in group I and in 17 patients (43.6%) in group II. Twelve patients (29.2%) in group I and 14 (35.9%) in group II underwent secondary CD.

The sequenced CT images, with respect to the images obtained at admission, showed that in group I, there was a predominance of “improved” CT scans (29 [70.7%]) (*p* ≤ 0.000), whereas in group II, only 16 (41%) showed this characteristic, with a difference between the groups of 29.7%.

When analyzing the “unchanged” CT scans, we found a higher frequency in group II, with a percentage difference between the groups of 21.4%. Regarding the “worse” CTs, there was also a higher frequency in group II but with a lower percentage difference of 8.3%. When applying the Mann–Whitney *U* test, we found that there was a significant difference between the groups.

Regarding complications, we found that there was a greater frequency of extraneurological complications in both groups, among which health care-related infections (HAIs) predominated, with 17 cases (41.5%) in group I and 30 patients (76.9%) (*p* ≤ 0.000) in group II, with a percentage difference between the groups of 35.4%. This difference in favor of group II was probably related to the longer length of stay of these patients and the longer time of AMV. Among the neurological complications, ICHT predominated in both groups, with 17 patients (41.5%) in group I and 25 patients (64.1%) (*p* ≤ 0.005) in group II, with a percentage difference between the groups of 22.6%. This was also the complication most related to deaths in both groups. When applying the Mann–Whitney *U* test, we found significant differences between the groups in high airway infections (*p* ≤ 0.005) and to a lesser extent with ICHT (*p* ≤ 0.07), in both cases with a predominance for group II.

**FIG. 4.**
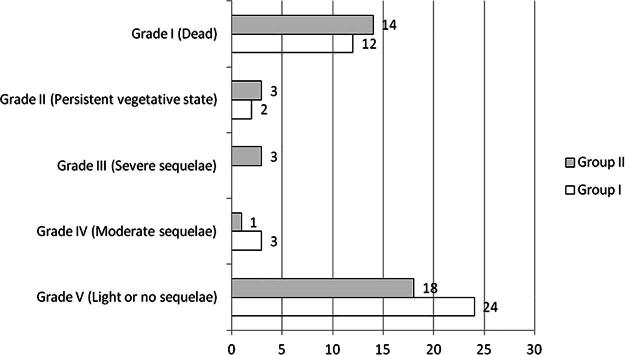
Result.

Figure 4 shows the results and shows how in group I, more than half of the patients, 24 (58.5%), were left with mild or no sequelae, with a mortality of 29.3%. In group II, 18 cases (46.1%) were left with mild or no sequelae, with a negative percentage difference of 12.4% compared with group I. In group II, 14 patients died (35.9%), for a percentage difference of 6.7% higher compared with group I. When applying the Pearson chi-square test for the status at discharge, with the categories discharged alive or deceased, there were no differences between the groups (*p* ≤ 0.694).

When we analyzed the results stratified into satisfactory (GOS between grades III–V) and unsatisfactory (grades I and II), we found that in group I, 27 (65.9%) had satisfactory results and 14 (34.1%) unsatisfactory, with a difference of 31.8% toward the predominance of satisfactory results. In group II, 17 patients (43.6%) had unsatisfactory results and 22 (56.4%) had satisfactory results, with a percentage difference in favor of satisfactory results of only 12.8%. The percentage difference in satisfactory results between the groups was 19% in favor of group I.

## Discussion

The real contribution of the different neuromonitoring variants in pediatric patients with sTBI is still controversial. Undoubtedly, ignoring the information offered by these methods in patients with severe traumatic head injury would be similar to asking a cardiologist to diagnose and treat a myocardial infarction without electrocardiographic monitoring, because patients undergoing MMN receive a personalized treatment modality, which allows early diagnosis of the state of heart disease and different intracranial variables, to optimize the treatment and diagnosis of complications, which facilitates their rapid solution. So its use is recommended in centers that have the required technology, which agrees with what has been reported by other authors.^26,27^ The publication of some studies such as BEST-TRIP and others,^26,28^ which have not been able to prove the benefit of using these techniques, adds controversy to the topic.

ICP was the main variable to be considered in this study. In group I, by having continuous monitoring, it was possible to quickly diagnose the control of this parameter and identify those in which high values persisted and trigger therapeutic escalation quickly. For the application of the algorithm without MMN in group II, the clinical and imaging criteria for the suspicion of ICHT were very important, but without the specificity provided by continuous ICP monitoring, it can be seen that all patients had criteria of elevated ICP in the first 24 h of admission and the diagnosis of its control was delayed, which shows the advantage of having MMN in terms of speed in obtaining diagnoses and quickly escalating to the therapeutic level; however, these findings did not show significant differences regarding the results.

In most of the studies reviewed, the threshold for the treatment of elevated ICP for all pediatric patients is recommended at 20 mmHg,^12^ a very controversial criterion, as neurophysiological variables that change with pediatric age are accepted nationally and internationally and which suggest that thresholds should be personalized for each age-group.^24^ Few attempts have been made to guide treatment in these patients with different thresholds for each age-group, as in the present study, the information available on this aspect is that no significant differences have been found in relation to the dichotomized results of the GOS 6 months after the trauma.^25,29^ No differences were found between the groups in this study either.

Currently, there is a tendency to consider that the quantitative value of ICP alone is not sufficient to initiate treatment in the event of ICHT;^30^ it is necessary to consider the qualitative element, the morphology of the pulse waves that they express the state of intracranial compliance. In the present study, this determination was made in group I but not in group II, and when the results were compared, there were no significant differences with regard to mortality. This information reinforces the already-established criterion that by controlling ICP, the pressures in the different intracranial compartments are balanced and in this way compliance improves. In those cases in which this objective is not achieved, the wave morphology with appearance of plateau waves or with confirmed plateau morphology, although the ICP figures are normal, they indicate the compromise of compliance and the need to adopt new diagnostic and therapeutic behaviors, which allow avoiding the development of uncontrollable ICHT. In the present study, DC was an alternative that facilitated the immediately reduced ICP levels in those who evidenced the presence of ICHT. DC converts the cranial cavity from a “closed home” to an “open home” and opens a therapeutic window first for evacuation of space-occupying lesions and rearrangement of structures and then diagnostic by serving to monitor intracranial structures through the use of transcraniectomy cranial ultrasound.

CPP was another important variable in the treatment of neurotraumatized pediatric patients. This was only calculated in group I, the work system planned for this study was based on the personalization of the treatment according to different age-groups,^25^ and finally the work team could not make comparisons between groups because the majority of the patients were between 5 and 17 years of age; however, data obtained in studies with a low level of evidence suggest maintaining values between 40 and 50 mmHg (level of evidence class III). They also state the subtlety that there may be specific thresholds for different age-groups, with values that could be below those recommended in younger children or higher than the range stated for older children,^12^ which is consistent with the concept of treatment for these patients that the authors defend in the present study,^25^ although this idea must be verified in subsequent studies.

In the neuromonitoring imaging of both groups, in addition to the elements usually considered in the admission CT, the measurement of the ONSD was very important, especially in group II, in which we found a greater frequently those with 3.5 mm or less, 21 (51.2%) in group I and 18 (46.2%) in group II. When relating the ONSD with the average ICP values in group I, it was striking how 24 (85.7%) (*p* ≤ 0.000) of 28 cases in the range between less than 3.5 mm and 5.5 mm had normal ICP values and 12 (92.3%) (*p* ≤ 0.000) of 13 children with diameters greater than 5.5 mm had high ICP values for their age. This finding could be a suggestion to start the treatment of ICHT when the ONSD is above 3.5 mm. Changes in ICP in the child cause changes in ONSD in seconds.^31^ Kayadibi et al.^32^ demonstrated the effectiveness of measuring the ONSD in children to estimate ICP by correlating it with CT findings through the Rotterdam Scale. Other authors agree on the correlation between ONSD and ICP in adults,^33,34^ so it is necessary to pay more attention to this variable in subsequent studies.

Neuroworsening occurred in 9 patients (22%) in group I, whereas in group II, there were 18 cases (46.1%), with a difference of 24.1% in favor of group II. This result differs from what was reported in the BEST-TRIP study,^28^ where there were no significant differences between the groups.

This finding is probably related to the absence of control and monitoring of intracranial parameters in group II, which influenced the late diagnosis of decompensated ICHT and the impossibility of using qualitative information on the status of intracranial compliance. This element can be solved with the estimation of ICP, CPP, and intracranial compliance in centers that have 24-h transcranial Doppler and other noninvasive neuromonitoring methods, which we do not have in our hospital.

The inclusion of these criteria in the algorithms was decisive for the indication of sequential imaging studies to investigate the appearance of new focal lesions or the worsening of existing ones and advance therapeutic escalation. In group I, as there was continuous monitoring of ICP and CPP, the appearance of these clinical signs could be suspected and prevented in some patients, which was not possible in group II.

The measures to control ICHT were organized depending on the needs to be treated and these did not always follow the planned order. Analgesia and sedation were used in all cases studied in both groups (100%), with the dual objective of guaranteeing patient–artificial ventilation machine synchrony and thus avoiding ICHT events. This was not considered in any of the algorithms as a direct measure to control ICP elevations but rather as a basic treatment. In both study groups, a high Therapeutic Intensive Level predominated, probably directly related to the number of patients who underwent primary DC; there were no significant differences between the groups. Generally, patients who have been subjected to an intense level of therapy show worse results;^35^ however, although in this study all the deceased had this level of therapy, 11 cases (47.8%) in group I and 17 (43.6%) in group II survived.

There was a higher frequency of extraneurological complications in both groups, among which HAIs predominated, with 17 cases (41.5%) in group I and 30 patients (76.9%) (*p* ≤ 0.000) in group II, with a percentage difference between the groups of 35.4%. This difference in favor of group II was probably related to the longer length of stay of these patients. Among the neurological complications, ICHT predominated in both groups, with 17 patients (41.5%) in group I and 25 patients (64.1%) (*p* ≤ 0.005) in group II, with a percentage difference between the groups of 22.6%. This was also the complication most related to deaths in both groups.

The results in this study did not show significant differences between the groups. This finding does not reject the importance of MMN; it is simply an argument that justifies the use of any of the two treatment variants and to a certain extent resolves an existing scientific and therapeutic contradiction for several years now,^28,36,37^ although, undoubtedly, patients whose treatment is established under the guidance of the MMN receive a personalized treatment modality, which allows an early diagnosis of the state of different intracranial variables, which optimizes the treatment and the diagnosis of complications, which facilitates their rapid solution, so its use is recommended in centers that have the required technology, which agrees with what was reported by other authors.^26,27^ The BEST-TRIP,^28^ a trial carried out in Latin America (Bolivia and Ecuador) in patients over 13 years of age, had two study arms, one with invasive ICP monitoring and the other with clinical and imaging monitoring. The report was carried out in 2012, and its authors concluded that, although there was a trend toward lower mortality and better care in patients with ICP monitoring, in terms of results, there were no differences between treatment modalities, which coincides with what was found in the present work. This study was highly criticized for its methodological limitations, and the mortality level in the trial was influenced by the prehospital management and the lack of rehabilitation after the ICU phase of treatment.

There are several elements to consider in this topic that should not be ignored. MMN does not impact mortality by itself; it is the therapeutic measures generated with the information obtained that can modify the natural evolution of the disease and influence this variable.^36,37^ Every patient with sTBI may or may not have MMN and must be subjected to treatment to control general and intracranial parameters such as the rise in ICP and the fall in CPP, phenomena that frequently occur in this type of patient. The use of a protocol, *per se*, can help to get better outcomes.

It is necessary to individualize treatments in sTBI because results differ between different GCS scores regardless of the use or not of MMN. In the treatment systems for these cases, novel elements must be considered from the anatomical, physiological, and interpretation points of view of the information offered by the type of neuromonitoring available, which may have an impact on the results.^30^ In conclusion, we can report that clinical and imaging monitoring was sufficient to guide treatment in the group without MMN, although the authors consider that MMN represents the ideal method to guide treatment in pediatric patients with sTBI.

There were no significant statistical differences between the groups regarding the results, but MMN showed advantages over clinical and imaging monitoring: the information provided facilitated the earlier identification of elevations in ICP and falls in CPP. It allowed combining the quantitative analysis with the qualitative analysis of the ICP. It guaranteed personalized treatment of patients according to their age-groups. Overall mortality was 6% lower in this group. The number of patients with mild or no sequelae was higher. The time of AMV was shorter. The stay in the PICU was shorter.

## Abbreviations Used

AMV: artificial mechanical ventilation
CBF: cerebral blood flow
CPP: caerebral perfusion pressure
CPPe: estimated cerebral perfusion pressure
CSF: cerebral spinal fluid
CT: computarized tomography
DBP: diastolic blood pressure
DC: decompressive craniectomy
GCS: glasgow coma scale
GOS: glasgow outcome scale
HAIs: healthcare related infections
ICHT: intracranial hypertension
ICP: intracranial pressure
Ka: argumentation coefficient
Kc: knowledge coefficient
Kcp: competition coefficient
MAP: mean arterial pressure
MMN: multimodal neuromonitoring
ONSD: optic nerve sheath diameter
PF: final pressure
PI: initial pressure
PVI: pressure-volume index
SBP: systolic blood pressure
sTBI: severe traumatic brain injury
TBI: traumatic brain injury
Vd: diastolic velocity
Vm: mean velocity
